# Infusion of the Leaves of the Ash-tree (Fraxinus Polygamie) in the Treatment of Gout and Rheumatism

**Published:** 1853-03

**Authors:** 


*Infusion of the leaves of the Ash-tree (Fraxinus polygamie) in the treatment of Gout and Rheumatism.* By Drs. POUGET and PEYRAUD. —The most useful acquisitions in materia medica are due generally to chance, or, rather, to a protective Providence over human health; *e.g.*, the antiperiodic properties of cinchona.

In 1842, Dr. Peyraud experienced his first attack of gout, for which he employed the usual remedies. The attack lasted twenty-five days. During the three following years the disease increased both in frequency and violence, and the patient went through the several forms of treatment without finding relief. He heard, by accident, that the infusion of the ash-leaves had long been considered a specific in the department of Dordogne, and that the peasantry of the district used it to "chase away their pains." Dr. Peyraud found it upon trial so useful that he had but one attack of the gout from 1845 to 1849.

M. B., a merchant of Bordeaux, was seized with an attack of gout, for the first time, in the right foot. He was ordered infusion of ash-leaves; upon the second day he was able to come down stairs and to leave his house. Ever since, he has had recourse to the same remedy with good effect upon experiencing premonitory symptoms, such as headache, loss of appetite, etc. The same success followed its administration in cases of acute articular rheumatism. A house-painter, aged 23, had, upon several occasions, suffered from acute articular rheumatism, accompanied by endocarditis, which prevented his following his occupation for four months or more. December 25, 1849, he had pain in the articulations of the feet, knees, and wrists, with swelling, accompanied by difficulty of respiration and inability to lie down. December 30, he was so weak that it was thought better, before bleeding him, to try the infusion of ash-leaves. In about twelve hours, and after the third dose, the patient was able to breathe easier, and there were fewer precordial pains. On the fourth day, the articular pains had diminished in intensity, the respiration was freer, and the appetite had returned. Jan. 22d.—He was able to resume work. The authors now administer it in the form of a fine powder in preference to the infusion. Other cases are related in the Essay.—*Op. cit.*, Nov. 1852.

